# Transcriptome and DNA methylation analysis reveals molecular mechanisms underlying intrahepatic cholangiocarcinoma progression

**DOI:** 10.1111/jcmm.16615

**Published:** 2021-05-19

**Authors:** Yuming Peng, Guohao Meng, Xinyi Sheng, Hongqiang Gao

**Affiliations:** ^1^ First Department of General Surgery Hunan Children’s Hospital Changsha China; ^2^ Department of Pathophysiology Key Laboratory of Cell Differentiation and Apoptosis of Chinese Ministry of Education Shanghai Jiaotong University School of Medicine Shanghai China

**Keywords:** DNA methylation, epigenetics, intrahepatic cholangiocarcinoma, progression, transcriptome, WGCNA

## Abstract

Intrahepatic cholangiocarcinoma (iCCA) is an aggressive malignancy with increasing incidence. It has been suggested that DNA methylation drives cancer development. However, the molecular mechanisms underlying iCCA progression and the roles of DNA methylation still remain elusive. In this study, weighted correlation networks were constructed to identify gene modules and hub genes associated with the tumour stage. We identified 12 gene modules, two of which were significantly positively or negatively related to the tumour stage， respectively. Key hub genes *SLC2A1*, *CDH3* and *EFHD2* showed increased expression across the tumour stage and were correlated with poor survival, whereas decrease of *FAM171A1*, *ONECUT1* and *PHYHIPL* was correlated with better survival. Pathway analysis revealed hedgehog pathway was activated in *CDH3* up‐regulated tumours, and chromosome separation was elevated in tumours expressing high *EFHD2*. JAK‐STAT pathway was overrepresented in *ONECUT1* down‐regulated tumours, whereas Rho GTPases‐formins signalling was activated in *PHYHIPL* down‐regulated tumours. Finally, significant negative associations between expression of *EFHD2*, *PHYHIPL* and promoter DNA methylation were detected, and alterations of DNA methylation were correlated with tumour survival. In summary, we identified key genes and pathways that may participate in progression of iCCA and proposed putative roles of DNA methylation in iCCA.

## 1 INTRODUCTION

Cholangiocarcinoma (CCA) constitutes a heterogeneous group of malignancies arising from the biliary tract, characterized by late diagnosis and rapid progression.^1^, ^2^ According to the anatomic location, CCA is categorized into distal CCA (dCCA), perihilar CCA (pCCA) and intrahepatic CCA (iCCA), which accounts for 25% of CCA.^3^, ^4^ iCCA is the second most common primary liver cancer after hepatocellular carcinoma, comprising approximately 15% of all primary hepatic malignancies.^1^ In general, the age‐standardized incidence for iCCA worldwide has been steadily increasing over the past few decades.^1^ iCCA is a highly aggressive malignancy whose 1‐ and 5‐year overall survival rates are approximately 30% and 18%, respectively.^4^ Complete surgical resection serves as the best treatment for long‐term survival for iCCA patients.^4^ For the patients to whom surgical resection is unamenable, the benefit of conventional chemotherapy and targeted therapy is limited.^5^ As a consequence, the mortality rates of iCCA are universally increasing.^6^ Despite recent applications of high‐throughput methods in the study of iCCA, the molecular pathogenesis and progression of the tumour still remain elusive.^7^ Therefore, it is urgent to advance our understanding of the molecular mechanisms driving progression of iCCA to improve patient welfare and outcome.

Weighted correlated network analysis, also known as weighted gene co‐expression analysis (WGCNA), functions as a biological meaningful data reduction approach.^8^ By relating modules to clinical traits, one can discover important gene modules with respect to clinical values. Intramodular genes with highest connectivity are considered critical hub genes, as they play central roles within the module. This method provides great opportunities to reveal important genes which drive the development of cancers.

DNA methylation is an epigenetic mechanism related to gene expression. DNA methylation of CpG islands (CGIs), which are DNA sequences enriched in CpG dinucleotides located at the promoter region, is associated with stable gene repression.^9^ In cancer, many tumour suppressor genes (TSGs) are silenced by DNA methylation.^10^ CCA displays many aberrant alterations of stereotypical cancer DNA methylome. Multi‐omics studies found that DNA methylation landscape underwent pervasive dysregulation with concomitant transcriptome alterations in iCCA.^11^, ^12^ DNA demethylation is compromised by *IDH1* and *IDH2* mutations at low to intermediate frequencies in iCCA, which are rarely detected in other subtypes of CCA.^2^ This leads to epigenome‐wide consequences, including increased 5‐methylcytosine (5mC) and decreased 5‐hydroxymethylcytosine (5hmC). Frequent genetic mutations of the key DNA methylation modulators indicate a significant impact of this epigenetic modification on iCCA. Notably, promoter hypermethylation and transcriptional silencing of TSGs are commonly described in CCA. The perturbed pathways include WNT, transforming growth factor (TGF)‐β, phosphoinositide 3‐kinase (PI3K) and NOTCH pathways.^2^, ^13^ More recently, transcriptional silencing of SOX17 by promoter hypermethylation was observed in CCA, resulting in inhibition of cholangiocyte differentiation and induction of oncogenes.^14^ Thus, hub genes for the iCCA development may be regulated by promoter DNA methylation.

Most transcriptome and DNA methylome analyses were performed on mixed CCA samples, which may not clarify the specific molecular profiles and underlying mechanisms of iCCA. That is because the molecular landscapes, particularly somatic mutations, largely differ between iCCA and other subtypes of CCA (dCCA and pCCA).^15^ In the current study, we constructed weighted correlated networks to identify key hub genes relevant to progression of iCCA. As a result, we identified *CDH3*, *EFHD2* as tumour‐promoting genes, and *ONECUT1*, *PHYHIPL* as tumour suppressing genes. We indicated their prognostic values in clinical practice. In addition, underlying signalling pathways, including hedgehog pathway, O‐glycan biosynthesis, JAK‐STAT pathway and Rho GTPases activating formins, were uncovered. Lastly, associations of key hub genes with promoter DNA hypermethylation were revealed.

## 2 MATERIALS AND METHODS

### 2.1 Data collection

The cholangiocarcinoma microarray data set was obtained from Gene Expression Omnibus (GEO) of the National Center for Biotechnology Information database (https://www.ncbi.nlm.nih.gov) with the accession code GSE89749. This data set was produced by the Illumina HumanHT‐12 V4.0 expression beadchip platform and included 118 cholangiocarcinoma samples and 2 normal samples. The corresponding DNA methylation data set GSE89803, which used the Illumina HumanMethylation450 BeadChip platform to profile DNA methylation in 138 tumour samples and 4 normal samples across approximately 450 000 CpGs genome‐wide, and associated array annotation data (accessible at https://support.illumina.com/) were obtained as well. For validation purpose, we also downloaded the GSE107943 data set, which profiled iCCA gene expression using next‐generation sequencing technique under the Illumina NextSeq 500 platform and contained 30 tumour samples and 27 adjacent normal liver samples.

### 2.2 Data pre‐processing

Different workflows were implemented to process two gene expression data sets. The limma R package^16^ released from the Bioconductor software project (https://bioconductor.org/) was used to process the raw data from GSE89749 as suggested. Specifically, background correction using negative controls, quantile normalization, log2 transformation and filtering out probes with detection *P*‐values of larger than 0.05 was performed consecutively. Probes were aggregated into genes by median for genes with multiple probes to obtain the gene‐level expression values. As the data set consisted of two batches, the combat function of the sva R package was used to correct for batch effect after processing data from two batches separately. Samples other than iCCA were removed, resulting in a sample of 83 tumours. For weighted correlation network construction, 43 samples from tumour stages I and IV were selected. Underexpressed genes were filtered out by variance and only the top 1/5 variable genes (5502 in total) were kept. Sample hierarchical clustering tree, which implemented the average linkage method, was constructed to identify outlier and finally 1 sample was found and removed. For the GSE107943 data set, gene filtering by variance was used to select the top 1/5 variable genes as previously mentioned. Subsequently, normalization was implemented with variance‐stabilizing transformation, using the DESeq2 R package.^17^ Finally, outlier detection by hierarchical clustering and outlier filtering was carried out, resulting in an expression matrix containing 11 555 genes and 30 samples.

### 2.3 Weighted correlation network construction

Weighted correlation network was constructed with the WGCNA R package to identify significant modules and hub genes related to iCCA progression as previously described.^18^ Firstly, the adjacency matrix a_i,j_ which measured the connection strength between two genes was calculated as follows:

$$a_{i,j}={\left|0.5+0.5\times cor\left(x_{i},x_{j}\right)\right|}^{\beta }$$

where x_i_ and x_j_ were vectors of gene expression values for genes i and j, respectively; β, also called power, was the soft threshold parameter, and Pearson correlation coefficient was used. The optimal β that resulted in approximate scale‐free topology as measured by the scale‐free topology fitting index R^2^ was selected. In the present study, *β* = 16 was selected since this was the local optimum where the corresponding *R* ^2^ reached 0.9. To better define gene co‐expression modules, topological overlap measure (TOM) was calculated on the basis of adjacency matrix as follows:

$${\mathrm{TOM}}_{i,j}=\frac{\sum_{u}a_{i,u}a_{u,j}+a_{i,j}}{min\left(k_{i},k_{j}\right)+1‐a_{i,j}}$$

where a denoted adjacency matrix as previously calculated and k denoted connectivity, that is, row sum of adjacencies.^19^ Subsequently, TOM‐based dissimilarity measure DistTOM_ij_ was obtained as 1‐TOM_ij_ and used for average linkage hierarchical clustering with the cutreeDynamic algorithm, where minimal module size was set at 30. Close modules as measured by correlation of module eigengenes were merged with a dissimilarity threshold of 0.3.

### 2.4 Identification of clinically significant modules and hub genes

Three key statistics were calculated for the identification of important modules and genes. First, module eigengene (ME) was defined as the first principal component of each module and served as an optimal summary of the gene expression profiles within a given module. The Pearson correlation coefficients between MEs and tumour stage were calculated to identify tumour progression–associated modules. Second, a quantitative measure of module membership (MM), also known as eigengene‐based connectivity (kME), was defined as the correlation between the ME of a given module and the expression profile of a gene. Finally, gene significance (GS) was calculated as the correlation between the gene expression and the clinical traits. Presumably, genes with higher GS and kME play more important roles in the given module and tumour progression. Therefore, genes with the absolute value of GS > 0.4 and kME > 0.4 in the modules were referred to as intramodular hub genes.

### 2.5 Gene ontology (GO) enrichment analysis

Gene ontology enrichment analysis was carried out in select modules using the EnrichGO function in the clusterProfiler R package as suggested.^20^ Three GO categories, including biological process (BP), cellular component (CC) and molecular function (MF), were involved in the analyses. The Benjamini‐Hochberg (BH) method was implemented to adjust for multiple comparisons and the resulting adjusted *P*‐values of < 0.05 represented statistically significant. For each category, the top 8 GO terms were retrieved.

### 2.6 Network visualization

The gene co‐expression networks of key modules were visualized with Cyotoscape 3.7.2. Network topological statistics were obtained using the Network Analyzer tool, and genes with the top 80 degree were selected for visualization. Hub genes were labelled in red, whereas other genes were labelled in module colours.

### 2.7 Gene set enrichment analysis (GSEA)

Gene set enrichment analysis was performed to investigate the mechanisms mediated by the hub genes in progression of tumour using the software GSEA 4.0.3 as suggested. For each hub gene, 82 valid tumours samples from GSE89749 were assigned to high expression (top 2/5 samples), low expression (bottom 2/5 samples) and median (median 1/5 samples) groups by expression of the specified gene. The KEGG and REACTOME canonical pathways of curated gene sets (C2), BP, MF and CC GO gene sets (C5) were used for GSEA. Parameters were set as default values. A nominal *P*‐value of < 0.05 was considered as statistically significant.

### 2.8 Identification of key DNA methylation alterations

The HumanMethylation450 BeadChip probes associated with hub genes were obtained according to the beadchip annotation. Gene expression profiles and beta values of DNA methylation were scaled, after which a permutation approach to linear regression was performed to study the relationship between gene expression and DNA methylation of the loci associated with the specified genes using the lmPerm R package with default parameters. The BH method was employed to correct for multiple comparisons and an adjusted *P*‐value of < 0.05 represented statistically significant. Probes with an absolute value of coefficient of > 0.3 and an adjusted *P*‐value of < 0.05 were retrieved.

### 2.9 Survival and other statistical analysis

Survival analysis of GSE89803 and GSE107943 data sets was performed using the Kaplan‐Meier method, and the statistical significance was determined by the log‐rank test, all of which were implemented with the survival and survminer packages. To characterize the expression changes of hub genes across the tumour stages (I, II, III and IV), the first principal component (PC1) of the identified hub genes was calculated and scaled. Subsequently, the differences among four stages were tested using the approximative k‐sample Fisher‐Pitman permutation test of the coin package. Comparisons of the select hub genes across the stages were carried out in a similar approach, that is, the gene expression profile was scaled and the k‐sample permutation test was performed afterwards. A *P*‐value of < 0.05 was considered as statistically significant. All the plots in this study except the co‐expression network plots were generated by R 3.6.2.

## 3 RESULTS

### 3.1 Construction of weighted correlation network in iCCA by WGCNA

**FIGURE 1:**
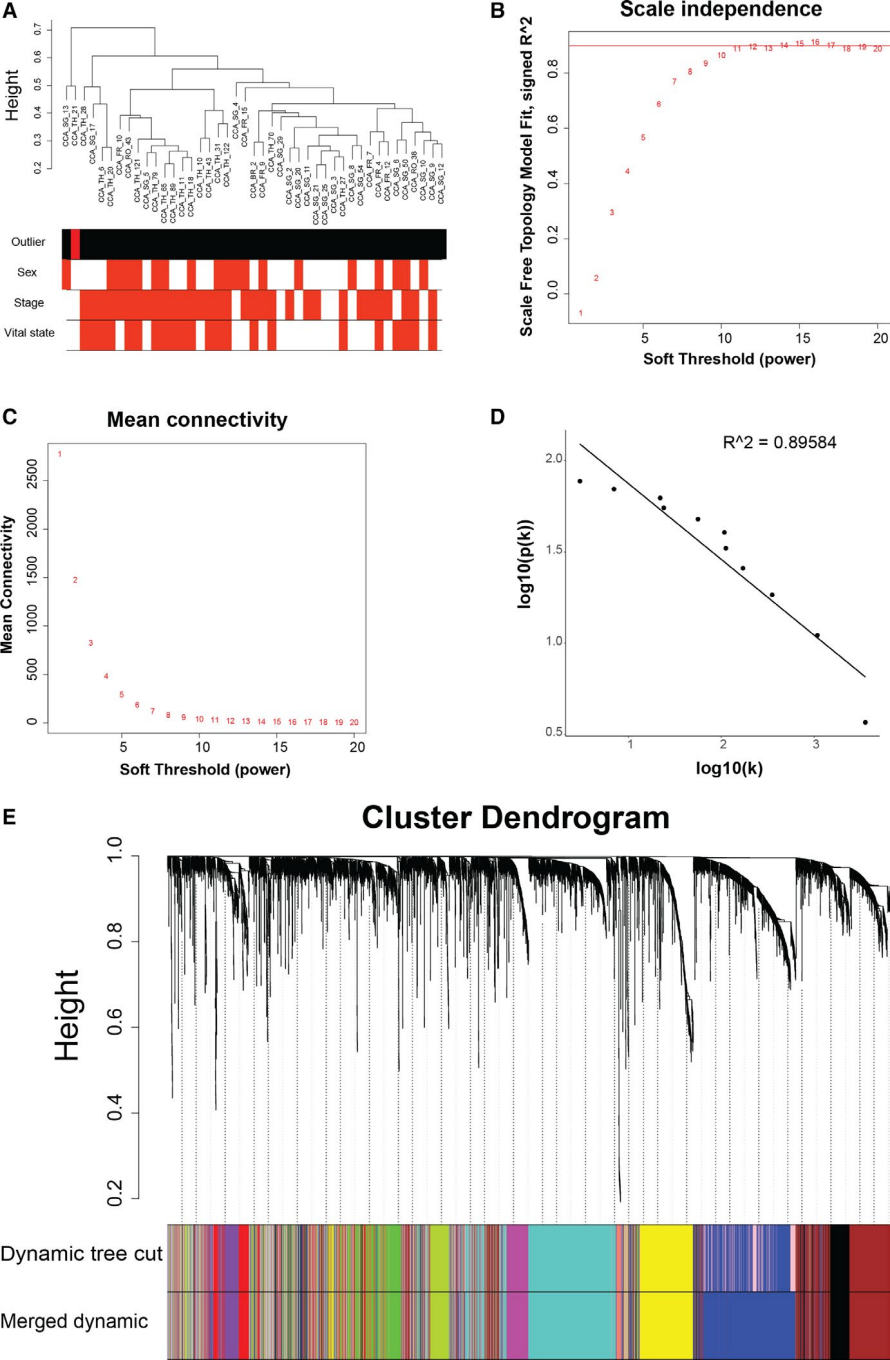
Weighted correlation network analysis identified twelve modules in intrahepatic cholangiocarcinoma. A, Sample cluster tree of intrahepatic cholangiocarcinoma samples. The colour bands underneath the tree represented whether the samples are outliers (in red), sex, tumour stage and vital state of the patients, respectively. B‐D, Scale‐free topology (SFT) criteria for choosing the power beta for the weighted correlation network. B, The SFT index as a function of different powers β. C, The mean connectivity as a function of powers β. D, Fitting linear model to log10(k) and log10(p(k)) when *β* = 16 was chosen (k denotes connectivity, and p(k) denotes frequency of k). E, Hierarchical cluster tree of the genes for analysis. The bands underneath the dendrogram show the gene modules detected with the dynamic tree cut algorithm and the merging procedure afterwards (each colour denotes one module). 12 gene modules were identified

Mircroarray gene expression profiles of normal and malignant bile duct samples coded as GSE89803 were obtained from GEO database.^21^ 83 iCCA samples were included for the study, among which 43 tumours staged I and IV were used for constructing gene co‐expression network. 5502 genes with the largest variance (top 1/5) across samples were kept. One outlying sample was detected with sample hierarchical clustering and then removed (Figure 1A). To determine the optimal power β that ensured scale‐free topology of the resulting network, the relationship between either scale independence or mean connectivity and power was analysed, respectively, and *β* = 16 was chosen (Figure 1B,C). Scale‐free topology of the network was supported by the goodness of fitting a linear model to log‐transformed connectivity k and frequency of k (p(k)), as was quantified by the fitting *R* ^2^ index (Figure 1D). We then constructed weighted correlation network by applying average linkage hierarchical clustering algorithm to dissimilarity measure of TOM and identified 12 gene modules after combining closely related modules (Figure 1E). The allocation of genes to each module is described in Table S1.

### 3.2 Relating module eigengenes to tumour stage identified iCCA progression‐associated modules

**FIGURE 2:**
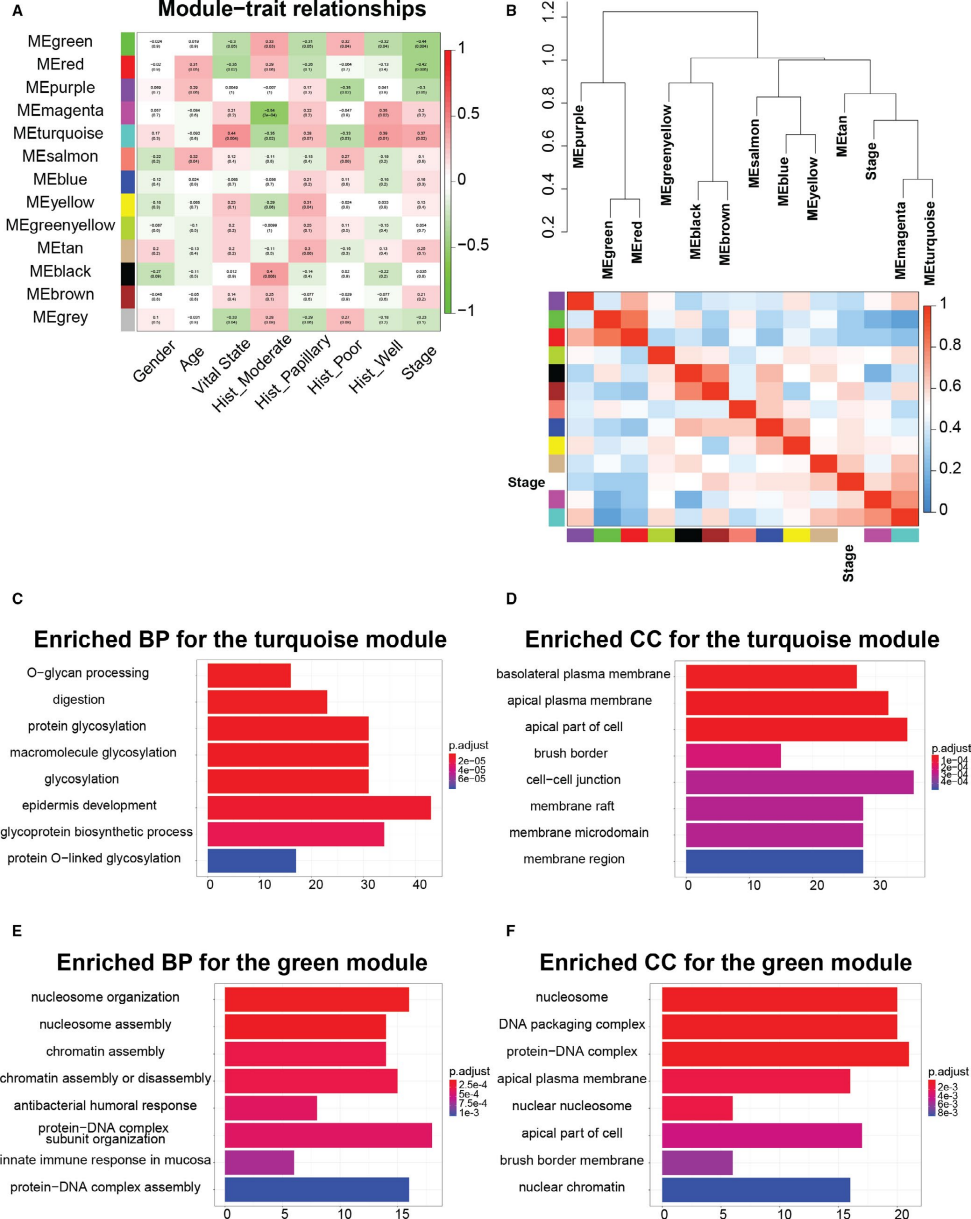
Identification of modules associated with progression of intrahepatic cholangiocarcinoma. A, Heatmap of the correlation between module eigengenes and select clinical traits of intrahepatic cholangiocarcinoma. B, Dendrogram and heatmap illustrating eigengene network representing the correlations among module eigengenes (MEs) and tumour stage. Hierarchical clustering was performed to group MEs of different modules and tumour stage, as is shown in the first row. The heatmap below shows the correlations among MEs and tumour stage. C and D, Gene ontology (GO) enrichment analysis of the turquoise module genes. Enriched biological process (BP) and cellular component (CC) terms are shown in (C and D), respectively. E and F, GO enrichment analysis of the green module genes. Enriched BP and CC terms are shown in (E and F), respectively. Top 8 GO terms with FDR < 0.05 are shown

To identify clinically significant gene modules, especially those related to the tumour stage of iCCA, we calculated ME of each module as the first principal component by principal component analysis (PCA), and correlated MEs to select clinical traits. The relationships between modules and clinical traits were illustrated by the heatmap, with correlation coefficients and corresponding *P*‐values shown (Figure 2A). We identified three modules that were significantly related to the tumour stage, including the green, turquoise and red modules. Among them, the green and red modules were negatively correlated with tumour stage, and the turquoise module was positively correlated with tumour stage and vital state, suggesting the underlying pathways of these modules can inhibit or promote tumour development. Dendrogram of MEs and correlation heatmap were plotted to describe the module relationship (Figure 2B). The turquoise and green modules were chosen for further investigation.

To understand the biological functions involved in turquoise and green modules, we examined enriched GO categories of them (Figure 2C‐F). The genes of turquoise module were mainly enriched in glycosylation‐related processes, epidermis development (Figure 2C), plasma membrane and cell‐cell junction (Figure 2D), whereas the genes of green module were mainly enriched in chromosome assembly–related processes, protein‐DNA interaction, innate immune response (Figure 2E), nucleosome and protein‐DNA complex (Figure 2F).

### 3.3 *SLC2A1*, *CDH3* and *EFHD2* of turquoise module were positively related to progression of iCCA

**FIGURE 3:**
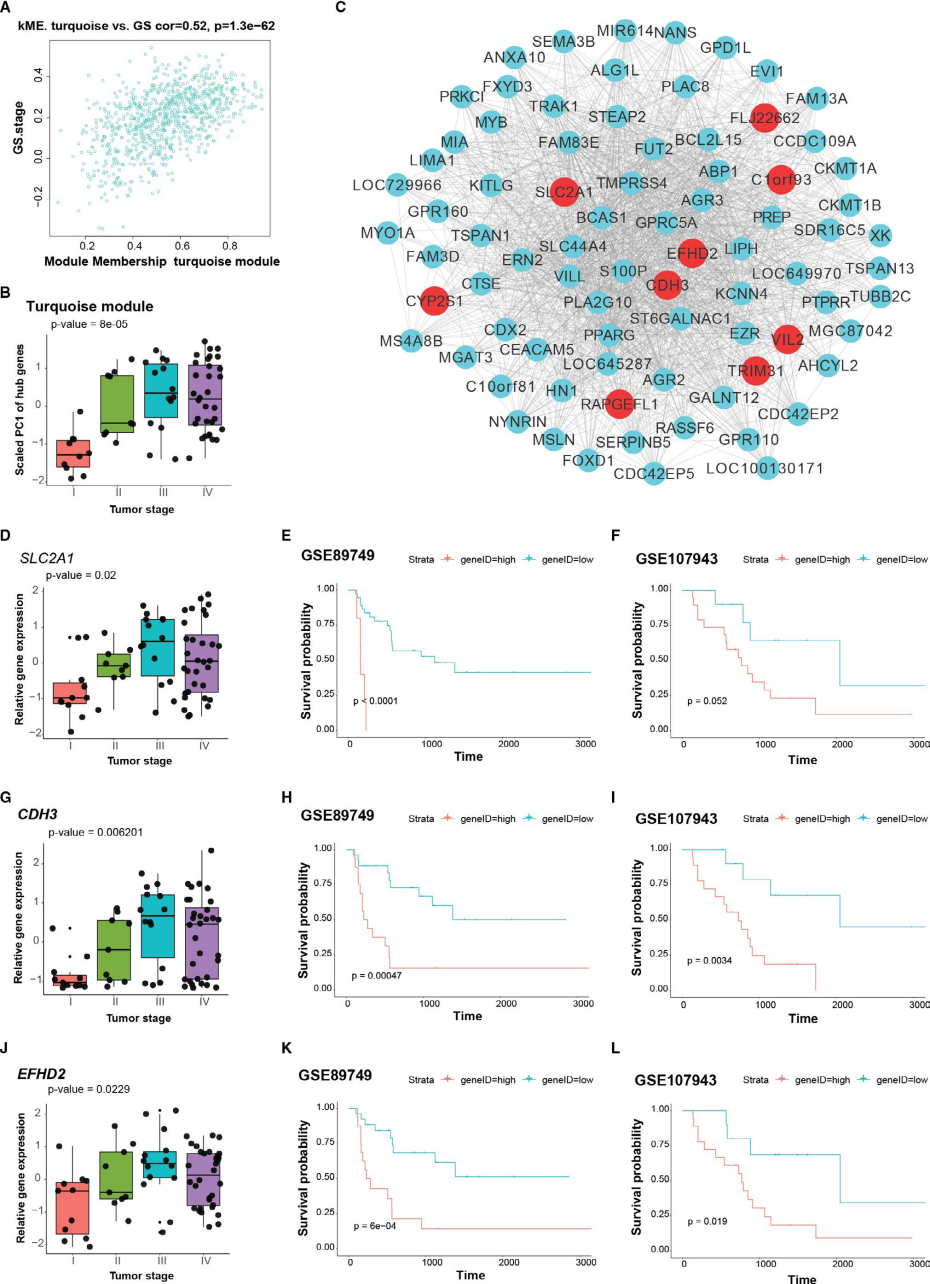
Intramodular analysis of the turquoise module. A, Scatter plot illustrating the relationship between gene significance (GS) of tumour stage and eigengene‐based connectivity (kME) of each gene in the turquoise module. Genes with absolute value of GS > 0.4 and kME > 0.4 were chose as hub genes. B, Bar plot shows the scaled first principal component (PC1) of hub genes in the turquoise module increased across tumour stage. C, Network of the turquoise module. Top 80 genes with largest connectivity as measured by degree are shown. Hub genes are labelled in red, and other genes are labelled in turquoise. D‐L, Clinical association of *SLC2A1*, *CDH3* and *EFHD2*. D and G, (J) Relative expression of *SLC2A1* (D), *CDH3* (G) and *EFHD2* (J) across tumour stage. E and H, (K) Kaplan‐Meier plots of GSE89749 show overall survival rates for the high and low expression groups of *SLC2A1* (E), *CDH3* (H) and *EFHD2* (K), respectively. (F,I and L) Kaplan‐Meier plots of GSE107943 show overall survival rates for the high and low expression groups of *SLC2A1* (F), *CDH3* (I) and *EFHD2* (L), respectively. *P* values in (B,D,G and J) were calculated by the k‐sample permutation test. *P* values shown in the Kaplan‐Meier plots were calculated by the log‐rank test

Focusing the analysis on gene modules and their highly connected intramodular hub genes serves as a biologically meaningful data reduction scheme.^8^ Additionally, these hub genes represent a small proportion of nodes with maximal information^18^; hence, we focused on hub genes for the network analysis. In the study, intramodular hub genes were referred to as those with absolute value of GS with respect to tumour stage of > 0.4 and kME of > 0.4. Within the turquoise module, the majority of genes were positively correlated with the tumour stage, and genes with higher GS had larger module membership (Figure 3A). Such significant correlations were also detected in the blue, brown, green‐yellow, magenta, purple, red, salmon, tan and yellow modules, but the magnitude of correlation was much smaller (Figure. S1). 45 hub genes were identified in the turquoise module (Table S2). Expression of the hub genes, which was represented as PC1, increased across the tumour stage (Figure 3B). We chose the top 80 genes of intramodular connectivity as measured in degree for visualization of the turquoise module, with hub genes highlighted in red (Figure 3C).

Among the hub genes, *SLC2A1*, *CDH3* and *EFHD2* displayed increased expression across the tumour stage (Figure 3D,G,J). Survival analysis by the Kaplan‐Meier method indicated that overexpression of these genes was related to smaller survival rates (Figure 3E,H,K), which were further confirmed by the GSE107943 iCCA data set (Figure 3F,I,L). Thus, these results demonstrate that the turquoise module may impact upon iCCA development, and *SLC2A1*, *CDH3* and *EFHD2* are iCCA prognosis relevant and may act as iCCA promoting genes.

### 3.4 *FAM171A1*, *ONECUT1* and *PHYHIPL* of green module were negatively related to progression of iC

**FIGURE 4:**
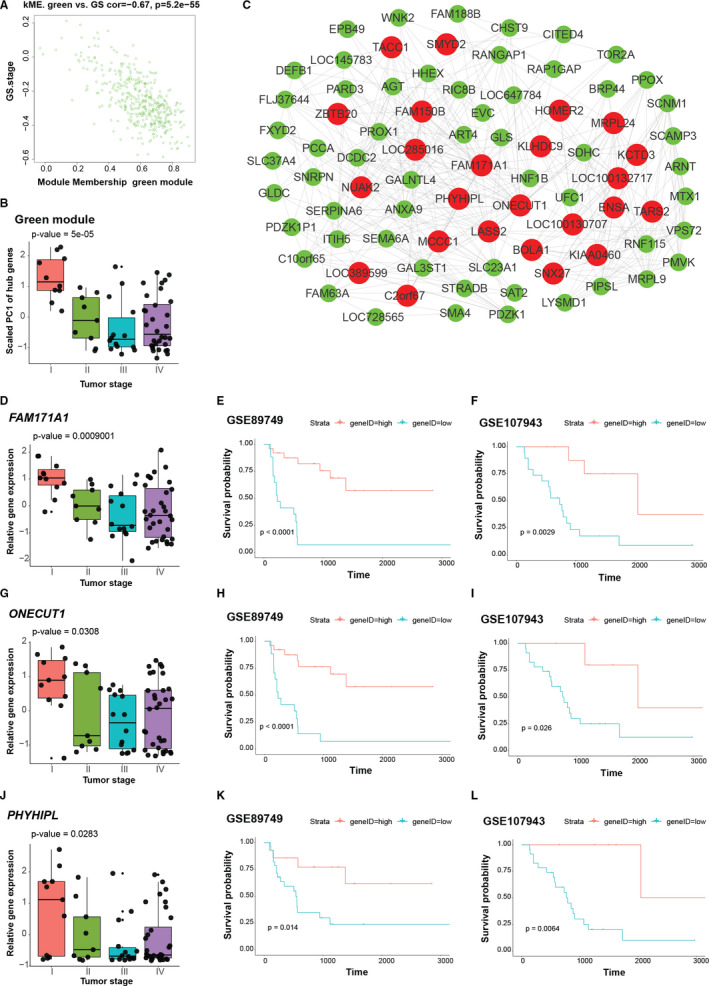
Intramodular analysis of the green module. A, Scatter plot illustrating the relationship between gene significance (GS) of tumour stage and eigengene‐based connectivity (kME) of each gene in the green module. Genes with absolute value of GS > 0.4 and kME > 0.4 were chose as hub genes. B, Bar plot shows the scaled first principal component (PC1) of hub genes in the green module increased across tumour stage. C, Network of the green module. Top 80 genes with largest connectivity as measured by degree are shown. Hub genes are labelled in red, and other genes are labelled in green. D‐L, Clinical association of *FAM171A1*, *ONECUT1* and *PHYHIPL*. (D,G and J) Relative expression of *FAM171A1* (D), *ONECUT1* (G) and *PHYHIPL* (J) across tumour stage. (E,H and K) Kaplan‐Meier plots of GSE89749 show overall survival rates for the high and low expression groups of *FAM171A1* (E), *ONECUT1* (H) and *PHYHIPL* (K), respectively. (F,I and L) Kaplan‐Meier plots of GSE107943 show overall survival rates for the high and low expression groups of *FAM171A1* (F), *ONECUT1* (I) and *PHYHIPL* (L), respectively. *P* values in (B,D and G) and (J) were calculated by the k‐sample permutation test. *P* values shown in the Kaplan‐Meier plots were calculated by the log‐rank test

Analysis of module‐trait relationships recognized the green module as an iCCA progression negatively correlated module. By plotting a scatterplot of GS for tumour stage against module membership, we found most genes of the green module were negatively related to the tumour stage and genes with larger absolute value of GS shared more module membership (Figure 4A). 52 genes were identified as hub genes in this module (Table S2). In contrast to the turquoise module, overall expression of the green module hub genes decreased across the tumour stage (Figure 4B). Top 80 genes of intramodular connectivity were visualized as a network with hub genes labelled in red (Figure 4C).

Consistent with the trend of overall expression of hub genes, expression of *FAM171A1*, *ONECUT1* and *PHYHIPL* decreased across the tumour stage (Figure 4D,G,J). The Kaplan‐Meier plots showed that patients with down‐regulation of these genes had smaller chances of survival (Figure 4E,H,K), which was further evidenced by the additional GSE107943 data set (Figure 4F,I,L). These observations imply that *FAM171A1*, *ONECUT1* and *PHYHIPL* are conversely related to iCCA progression and may have tumour suppressing effect.

### 3.5 Identification of key biological processes and pathways of hub genes underlying iCCA progression

**FIGURE 5:**
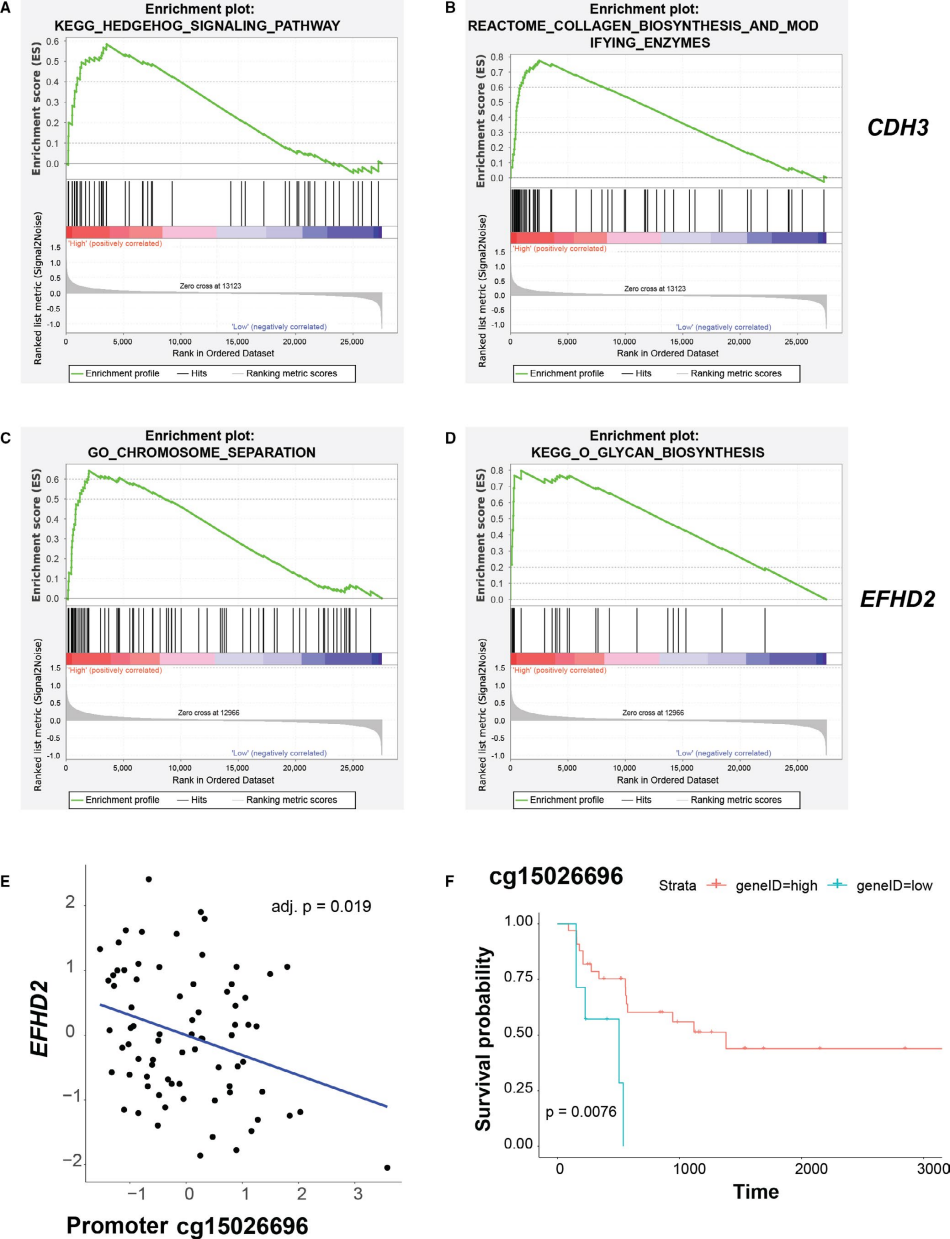
Molecular mechanism associated with *CDH3* and *EFHD2* of turquoise module underlying progression of intrahepatic cholangiocarcinoma. (A and B) Gene set enrichment analysis (GSEA) revealed biological processes and pathways associated with *CDH3* underlying progression of intrahepatic cholangiocarcinoma (iCCA). iCCA samples with high (top 2/5) or low (bottom 2/5) expression of the hub gene were assigned to two groups (high or low expression groups) and used for GSEA. Enrichment score (ES) on the y‐axis measures the degree to which a given gene set is overrepresented in either of the two groups. Gene sets with a nominal *P*‐value of < 0.05 were chose. (C and D) Gene set enrichment analysis (GSEA) revealed biological processes and pathways associated with *EFHD2* underlying progression of iCCA. E, Negative correlation between DNA methylation probe cg15026696 and expression of *EFHD2*. F, Kaplan‐Meier plots of GSE89749 showed overall survival rates for the high and low DNA methylation groups of cg15026696. For the correlation of gene expression and DNA methylation, the BH method was performed to adjust for multiple comparisons

To investigate potential mechanism of the hub genes, we performed GSEA to examine enriched biological processes and signalling pathways for *CDH3* and *EFHD2* of the turquoise module. We found that hedgehog signalling pathway and collagen biosynthesis and modifying enzymes were overrepresented in *CDH3* high expression samples (Figure 5A,B). Indeed, hedgehog pathway was suggested to be participated in cancer development and invasiveness.^22^ For *EFHD2*, chromosome separation and O‐glycan biosynthesis were found to be enriched in *EFHD2* high expression group (Figure 5C,D). It is implicated that O‐glycan biosynthesis plays a role in cancer development and invasion.^23^ Taken together, these findings indicate that *CDH3* and *EFHD2* can activate these pathways to promote tumour progression.

**FIGURE 6:**
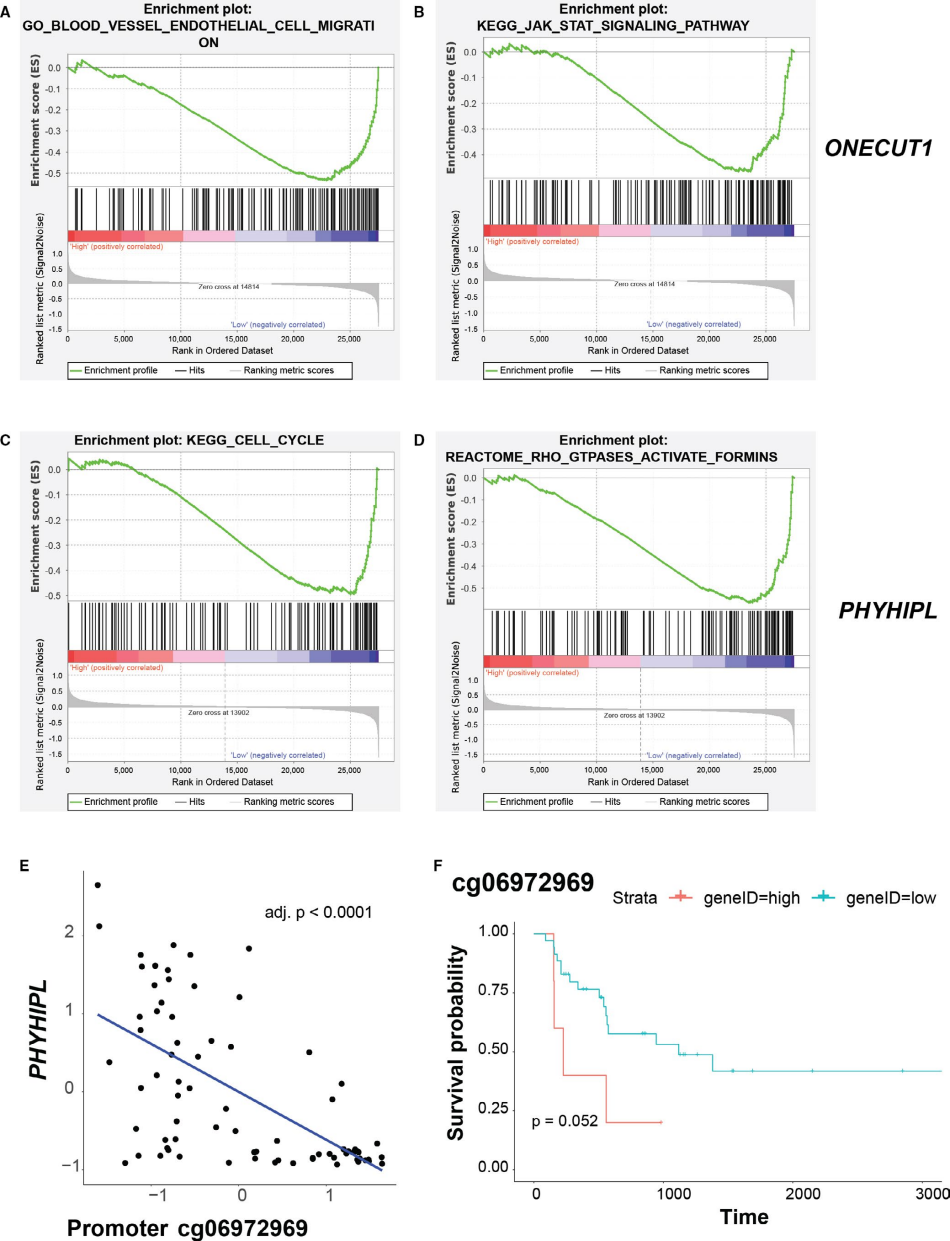
Molecular mechanism associated with *ONECUT1* and *PHYHIPL* of green module underlying progression of intrahepatic cholangiocarcinoma. A and B, Gene set enrichment analysis (GSEA) revealed biological processes and pathways associated with *ONECUT1* underlying progression of intrahepatic cholangiocarcinoma (iCCA). iCCA samples with high (top 2/5) or low (bottom 2/5) expression of the hub gene were assigned to two groups (high or low expression groups) and used for GSEA. Enrichment score (ES) on the y‐axis measures the degree to which a given gene set is overrepresented in either of the two groups. Gene sets with a nominal *P*‐value of < 0.05 were chose. C and D, Gene set enrichment analysis (GSEA) revealed biological processes and pathways associated with *PHYHIPL* underlying progression of iCCA. E, Negative correlation between DNA methylation probe cg06972969 and expression of *PHYHIPL*. F, Kaplan‐Meier plots of GSE89749 showed overall survival rates for the high and low DNA methylation groups of cg06972969. For the correlation of gene expression and DNA methylation, the BH method was performed to adjust for multiple comparisons

We applied similar strategies to *ONECUT1* and *PHYHIPL* of the green module. Blood vessel endothelial cell migration and JAK‐STAT signalling pathway were overrepresented in *ONECUT1* down‐regulated samples (Figure 6A,B), whereas cell cycle and Rho GTPases activating formins were enriched in *PHYHIPL* down‐regulated samples (Figure 6C,D). Given that activation of these pathways were involved in tumour development,^24^, ^25^, ^26^ these observations indicate that down‐regulation of *ONECUT1* and *PHYHIPL* can induce activation of these key pathways to promote iCCA progression. Detailed statistics of GSEA can be found in Table S3.

### 3.6 DNA methylation was involved in the regulation of key hub genes

We speculated dysregulation of the hub genes may be associated with aberrant DNA methylation in promoter regions. Thus, we examined the relationships between DNA methylation and mRNA expression and found that hypermethylation of promoter loci cg15026696 and cg06972969 were associated with decreased expression of *EFHD2* (Figure 5E) and *PHYHIPL* (Figure 6E), respectively. To further investigate the clinical significance of these loci, we conducted survival analysis of the DNA methylation loci and found that the hypomethylation of cg15026696 (Figure 5F) showed significant lower survival rates, whereas hypermethylation of cg06972969 (Figure 6F) was correlated with significant lower survival rates.

## 4 DISCUSSION

Intrahepatic cholangiocarcinoma has become a global burden to public health because of its increasing incidence and poor prognosis.^1^, ^4^ Currently, there is little treatment option with proven benefit for the tumour at advanced stage.^5^ Thus, it is urgently needed to explore the molecular mechanisms of iCCA progression and identify targets for therapeutics. In the present study, we systematically investigated gene co‐expression pattern across different stages of iCCA using WGCNA and detected 12 gene modules, among which two modules (the turquoise and green modules) were positively or negatively related to tumour development, respectively. Intramodular analysis identified *CDH3*, *EFHD2* (turquoise), *ONECUT1* and *PHYHIPL* (green) as key module hub genes. Additionally, signalling pathways and biological processes including hedgehog pathway, O‐glycan biosynthesis, JAK‐STAT pathway and Rho GTPases activating formins were revealed to be involved in iCCA development mediated by the hub genes. DNA methylation of certain promoter loci was significantly correlated with expression level of the hub genes and had prognostic value for survival of patients, indicative of the potential roles of epigenetic impact on iCCA progression.

The turquoise module, enriched in protein glycosylation and epidermis development, was linked to advanced stage of iCCA. Overall expression of the hub genes was increased across tumour stages, indicating that the hub genes were related to the tumour progression. Among the hub genes, *SLC2A1*, *CDH3* and *EFHD2* increased across tumour stages and were associated with poorer survival probability, suggesting their critical roles in iCCA. In line with our findings, higher expression of *SLC2A1*, which is a protein coding gene for glucose transporter protein type 1 (GLUT1), is associated with aggressive behaviour of iCCA and poor prognosis.^27^ Furthermore, up‐regulation of GLUT1 leads to growth of CCA and cell migration.^28^ Cadherin 3, also known as P‐Cadherin and encoded by *CDH3*, functions as a cell‐cell adhesion protein. Aberrant expression of Cadherin 3 has been described in many carcinoma and sarcoma, including CCA.^29^ Its role in cancer was further supported by the study showing Cadherin 3 increased the mobility of cancer cells.^30^ EFHD2, whose function in malignancies remained unexplored, was reported to stimulate tumour invasion and metastasis.^31^ These data indicate that these hub genes may participate in invasion and development of iCCA and highlight putative novel role of EFHD2 in iCCA, but functional experiment was needed to further elucidate their biological significance.

In contrast, the green module was inversely correlated with iCCA stage. It was enriched in chromatin and nucleosome organization. Dysfunction of the hub genes of the module may stimulate iCCA invasion, as was indicated by the finding that overall expression of the hub genes decreased across the tumour stage. Among them, *FAM171A1*, *ONCUT1* and *PHYHIPL* were identified as key hub genes. FAM171A1, also known as astroprincin (APTN), was reported to overexpress in brain astrocytes and involve in regulation of cytoskeletal dynamics, hence the cell shape and growth of cancer cells.^32^ Intriguingly, expression of FAM171A1 increased in triple‐negative breast tumour (TNBC) but decreased in non‐TNBC compared to normal tissue,^33^ whereas its role and significance in iCCA or CCA remains unclear. ONCUT1 is a transcription factor that is enriched in the liver and stimulates liver‐expressed genes. Previous study suggested that ONCUT1 was down‐regulated in HCC and acted as tumour suppressing gene in cell line experiment.^34^ Likewise, ONCUT1 expression was lost in pancreatic cancer cells, and its up‐regulation resulted in a reduction of invasiveness.^35^ Considering the close relationship of origins of these tumours, ONCUT1 may be involved in decrease of iCCA invasiveness. Biological function of PHYHIPL remains to be elucidated, probably related to central nervous system development. In the present study, down‐regulation of these hub genes was observed across the tumour stage of iCCA, which was accompanied by poor survival probability. Our study identified novel iCCA‐associated genes *FAM171A1*, *ONCUT1* and *PHYHIPL* and implied their tumour suppressing roles and prognosis values.

Further analysis revealed novel biological processes and signalling pathways mediated by CDH3 and EFHD2 of the turquoise module. Hedgehog signalling pathway governs complex developmental processes including cell proliferation. Aberrant activation of hedgehog pathway drives tumour initiation and maintenance.^36^ Increasing evidence implicated dysfunction of hedgehog signalling in initiation and progression of various cancers, including breast, pancreatic and hepatocellular cancers. In particular, two inhibitors of hedgehog pathway have been approved to treat basal cell carcinoma and medulloblastoma.^22^ Collagen fibre organization is implicated in cancer metastasis. Prior study suggested CDH3 promoted the activation of β‐Pix/CDC42 axis through collagen fibre orientation to facilitate directional collective cell migration, which was crucial for cancer metastasis.^37^ Based on the GSEA results, we postulate that CDH3 promotes iCCA invasion through activation of hedgehog signalling pathway or modulating collagen fibre organization. Our GSEA findings related chromosome separation and O‐glycan biosynthesis to EFHD2. Chromosomal instability is a hallmark of tumour that results from chromosome separation errors during mitosis, and it is correlated with poor prognosis, metastasis and therapeutic resistance.^38^ More importantly, errors in chromosome separation spills DNA into the cytosol, leading to activation of the cGAS‐STING cytosolic DNA‐sensing pathway and therefore promoting tumour invasion and metastasis.^39^ O‐glycosylation is a covalent post‐translational modification of protein. The tumour‐related O‐glycan Tn antigen is highly expressed in many tumours, including breast, lung, colon, bladder and stomach tumours, and its presence is related to metastasis and poor progonosis.^40^ It was reported that high levels of O‐glycan Tn and sTn antigen promoted growth and metastasis of breast tumour in vivo.^41^ In addition, T antigen regulated adhesion of tumour cells to the endothelium by galectin‐3, driving metastasis.^40^ We postulate that EFHD2 acts as tumour‐promoting gene probably through inducing chromosome separation errors or O‐glycan modification of key adhesion proteins and proteases that are associated with metastasis. Collectively, we indicate hedgehog signalling pathway and collagen fibre organization are critical pathways for CDH3, whereas aberrant chromosome separation and O‐glycan biosynthesis serve as mechanisms for EFHD2, regarding driving iCCA progression.

Moreover, our findings revealed key pathways mediated by suppression of ONECUT1 and PHYHIPL for the tumour progression. Blood vessel endothelial cell migration is involved in angiogenesis, which is required for tumour growth.^42^ Activation of JAK‐STAT signalling exerts effect on tumour survival, proliferation and invasion and has been recognized as drug targets in many cancers, including blood, breast, prostate and brain cancers. For instance, STAT3 activation acts as a crucial regulator of gliomagenesis by inducing angiogenesis, immunosuppression and tumour invasion.^25^ We speculate that suppression of ONECUT1 promotes growth and invasion of iCCA by regulating angiogenesis or JAK‐STAT pathway, but the detailed relationship remains to be studied. Cell cycle of human cells is governed by essential pathways including cyclin D‐CDK4/6‐Rb pathway. Its crucial role in cancer is highlighted by the findings that nearly all tumours possess alterations in a component and that changes in upstream tumour suppressors and oncoproteins may function by influencing cell cycle.^43^ Invasive cell migration is a critical step for tumour metastasis and involves Rho GTPase–regulated reorganization of actin cytoskeleton. Rho GTPase functions via downstream effectors, one of which is the formins family. Suppression of formin‐like 2 (FMNL2) inhibits RhoC‐dependent invasive cell migration.^26^ Our data indicate inhibition of PHYHIPL suppresses iCCA invasion through influencing cell cycle activity or Rho GTPase‐formins pathway. This is the first report that establishes the relationships of these pathways with ONECUT1 and PHYHIPL in iCCA.

Our study further evaluated the associations of DNA methylation with expression of the select hub genes. We detected significantly negative correlation between gene expression and DNA methylation in promoter regions of *EFHD2* and *PHYHIPL* in iCCA. Remarkably, these DNA methylation loci predicted survival of the patients, suggesting the impact of DNA methylation on iCCA development. Substantial evidence indicates a critical role of DNA methylation dysregulation in iCCA. For instance, *IDH* mutations at hotspots acquire gain‐of‐function activity and produce 2‐hydroxyglutarate (2‐HG) in CCA, which impairs DNA demethylation mediated by TET2, and thus result in evidently aberrant 5mC pattern and concomitant down‐regulated chromatin modifiers.^13^, ^44^ Another study revealed that CCA patients with high rate of *IDH* mutation and CpG shore hypermethylation had better prognosis.^21^ Furthermore, transcriptional repression of classic TSGs putatively mediated by promoter hypermethylation was reported. Such examples include genes involved in WNT, TGF‐β and PI3K pathways.^2^ Relevantly, this inhibitory effect was revealed in the context of microRNA in iCCA, as was shown by a recent study reporting that aberrant promoter hypermethylation‐induced suppression of miR‐212‐3p led to overexpression of MUC13 in iCCA and metastasis via the EGFR/PI3K/AKT pathway.^45^ Thus, our findings provide meaningful clues for novel examples of such dependency in iCCA progression. Notably, although early studies provided substantial evidence correlating high levels of promoter methylation with transcriptional silencing, more and more examples identify contexts where this observation does not always remain true.^46^ For instance, high levels of promoter methylation correlated with active expression of *EBF3*, *MGMT*, *HOXD12* and *GATA4* in melanoma, indicating other factors may affect the relations of DNA methylation with transcriptional activity.^47^ Thus, biological assays should be performed to investigate if DNA methylation has regulatory effect on transcriptional activities in iCCA.

Apart from the limitations discussed, this study did not apply multivariate survival model to account for known effects, mainly due to insufficient samples in the data set. With the application of whole genome DNA methylation profiling techniques, it will become feasible in the future. In summary, our report characterized molecular signature related to iCCA progression identified novel genes that may participate in promoting or inhibiting invasion and development of the tumour. Putative biological processes, signalling pathways of these genes and DNA methylation relations were revealed. These findings provide essential insights in understanding the biology of iCCA development.

## 5 ETHICAL APPROVAL AND CONSENT TO PARTICIPATE

Not applicable.

## CONFLICT OF INTEREST

The authors declare that they have no competing interests.

## AUTHOR CONTRIBUTION

**Yuming Peng:** Data curation (lead); Formal analysis (lead); Funding acquisition (lead); Methodology (lead); Visualization (lead); Writing‐original draft (lead); Writing‐review & editing (supporting). **Guohao Meng:** Conceptualization (lead); Data curation (supporting); Formal analysis (equal); Methodology (supporting); Project administration (lead); Supervision (lead); Visualization (equal); Writing‐original draft (equal); Writing‐review & editing (lead). **Xinyi Sheng:** Data curation (supporting); Formal analysis (supporting); Methodology (supporting); Visualization (supporting); Writing‐review & editing (supporting). **Hongqiang Gao:** Data curation (supporting); Visualization (supporting); Writing‐review & editing (supporting).

## Supporting information

Table S1

Table S2

Table S3

## Data Availability

All data required for evaluating the conclusions are present in the paper and supplementary materials. The data sets used the study can be accessed from GEO database with accession code GSE89749, GSE89803 and GSE107943.

## References

[1] Banales JM , Marin JJG , Lamarca A , et al. Cholangiocarcinoma 2020: the next horizon in mechanisms and management. Nat Rev Gastroenterol Hepatol. 2020;17(9):557‐588. 10.1038/s41575-020-0310-z 32606456 PMC7447603

[2] O’Rourke CJ , Lafuente‐Barquero J , Andersen JB . Epigenome remodeling in cholangiocarcinoma. Trends Cancer. 2019;5(6):335‐350. 10.1016/j.trecan.2019.05.002 31208696

[3] Banales JM , Cardinale V , Carpino G , et al. Expert consensus document: cholangiocarcinoma: current knowledge and future perspectives consensus statement from the european network for the study of cholangiocarcinoma (ENS‐CCA). Nat Rev Gastroenterol Hepatol. 2016;13(5):261‐280. 10.1038/nrgastro.2016.51 27095655

[4] El‐Diwany R , Pawlik TM , Ejaz A . Intrahepatic cholangiocarcinoma. Surg Oncol Clin N Am. 2019;28(4):587‐599. 10.1016/j.soc.2019.06.002 31472907

[5] Shiao M‐S , Chiablaem K , Charoensawan V , Ngamphaiboon N , Jinawath N . Emergence of intrahepatic cholangiocarcinoma: how high‐throughput technologies expedite the solutions for a rare cancer type. Front Genet. 2018;9:309. 10.3389/fgene.2018.00309 30158952 PMC6104394

[6] Khan SA , Genus T , Morement H , Murphy A , Rous B , Tataru D . Global trends in mortality from intrahepatic and extrahepatic cholangiocarcinoma. J Hepatol. 2019;71(6):1261‐1262. 10.1016/j.jhep.2019.07.024 31558288

[7] Braconi C , Roessler S , Kruk B , Lammert F , Krawczyk M , Andersen JB . Molecular perturbations in cholangiocarcinoma: is it time for precision medicine? Liver Int. 2019;39(S1):32‐42. 10.1111/liv.14085 30829432

[8] Levine AJ , Miller JA , Shapshak P , et al. Systems analysis of human brain gene expression: mechanisms for HIV‐associated neurocognitive impairment and common pathways with Alzheimer’s disease. BMC Med Genomics. 2013;6:4. 10.1186/1755-8794-6-4 23406646 PMC3626801

[9] Luo C , Hajkova P , Ecker JR . Dynamic DNA methylation: In the right place at the right time. Science. 2018;361(6409):1336‐1340. 10.1126/science.aat6806 30262495 PMC6197482

[10] Michalak EM , Burr ML , Bannister AJ , Dawson MA . The roles of DNA, RNA and histone methylation in ageing and cancer. Nat Rev Mol Cell Biol. 2019;20(10):573‐589. 10.1038/s41580-019-0143-1 31270442

[11] Goeppert B , Toth R , Singer S , et al. Integrative analysis defines distinct prognostic subgroups of intrahepatic cholangiocarcinoma. Hepatology. 2019;69(5):2091‐2106. 10.1002/hep.30493 30615206 PMC6594081

[12] Farshidfar F , Zheng S , Gingras M‐C , et al. Integrative genomic analysis of cholangiocarcinoma identifies distinct IDH‐mutant molecular profiles. Cell Rep. 2017;18(11):2780‐2794. 10.1016/j.celrep.2017.02.033 28297679 PMC5493145

[13] O’Rourke CJ , Munoz‐Garrido P , Aguayo EL , Andersen JB . Epigenome dysregulation in cholangiocarcinoma. Biochim Biophys Acta Mol Basis Dis. 2018;1864(4):1423‐1434. 10.1016/j.bbadis.2017.06.014 28645654

[14] Merino‐Azpitarte M , Lozano E , Perugorria MJ , et al. SOX17 regulates cholangiocyte differentiation and acts as a tumor suppressor in cholangiocarcinoma. J Hepatol. 2017;67(1):72‐83. 10.1016/j.jhep.2017.02.017 28237397 PMC5502751

[15] Lowery MA , Ptashkin R , Jordan E , et al. comprehensive molecular profiling of intrahepatic and extrahepatic cholangiocarcinomas: potential targets for intervention. Clin Cancer Res. 2018;24(17):4154‐4161. 10.1158/1078-0432.CCR-18-0078 29848569 PMC6642361

[16] Ritchie ME , Phipson B , Wu D , et al. limma powers differential expression analyses for RNA‐sequencing and microarray studies. Nucleic Acids Res. 2015;43(7):e47. 10.1093/nar/gkv007 25605792 PMC4402510

[17] Love MI , Huber W , Anders S . Moderated estimation of fold change and dispersion for RNA‐seq data with DESeq2. Genome Biol. 2014;15(12):550. 10.1186/s13059-014-0550-8 25516281 PMC4302049

[18] Langfelder P , Horvath S . WGCNA: an R package for weighted correlation network analysis. BMC Bioinformatics. 2008;9:559. 10.1186/1471-2105-9-559 19114008 PMC2631488

[19] Song L , Langfelder P , Horvath S . Comparison of co‐expression measures: mutual information, correlation, and model based indices. BMC Bioinformatics. 2012;13:328. 10.1186/1471-2105-13-328 23217028 PMC3586947

[20] Yu G , Wang L‐G , Han Y , He Q‐Y . clusterProfiler: an R package for comparing biological themes among gene clusters. OMICS. 2012;16(5):284‐287. 10.1089/omi.2011.0118 22455463 PMC3339379

[21] Jusakul A , Cutcutache I , Yong CH , et al. Whole‐genome and epigenomic landscapes of etiologically distinct subtypes of cholangiocarcinoma. Cancer Discov. 2017;7(10):1116‐1135. 10.1158/2159-8290.CD-17-0368 28667006 PMC5628134

[22] Xu Y , Song S , Wang Z , Ajani JA . The role of hedgehog signaling in gastric cancer: molecular mechanisms, clinical potential, and perspective. Cell Commun Signal. 2019;17(1):157. 10.1186/s12964-019-0479-3 31775795 PMC6882007

[23] Oliveira‐Ferrer L , Legler K , Milde‐Langosch K . Role of protein glycosylation in cancer metastasis. Semin Cancer Biol. 2017;44:141‐152. 10.1016/j.semcancer.2017.03.002 28315783

[24] De Palma M , Biziato D , Petrova TV . Microenvironmental regulation of tumour angiogenesis. Nat Rev Cancer. 2017;17(8):457‐474. 10.1038/nrc.2017.51 28706266

[25] Pencik J , Pham HTT , Schmoellerl J , et al. JAK‐STAT signaling in cancer: from cytokines to non‐coding genome. Cytokine. 2016;87:26‐36. 10.1016/j.cyto.2016.06.017 27349799 PMC6059362

[26] Kitzing TM , Wang Y , Pertz O , Copeland JW , Grosse R . Formin‐like 2 drives amoeboid invasive cell motility downstream of RhoC. Oncogene. 2010;29(16):2441‐2448. 10.1038/onc.2009.515 20101212

[27] Kubo Y , Aishima S , Tanaka Y , et al. Different expression of glucose transporters in the progression of intrahepatic cholangiocarcinoma. Hum Pathol. 2014;45(8):1610‐1617. 10.1016/j.humpath.2014.03.008 24824030

[28] Labib PL , Goodchild G , Pereira SP . Molecular pathogenesis of cholangiocarcinoma. BMC Cancer. 2019;19(1):185. 10.1186/s12885-019-5391-0 30819129 PMC6394015

[29] Plutoni C , Bazellieres E , Le Borgne‐Rochet M , et al. P‐cadherin promotes collective cell migration via a Cdc42‐mediated increase in mechanical forces. J Cell Biol. 2016;212(2):199‐217. 10.1083/jcb.201505105 26783302 PMC4738379

[30] Taniuchi K , Nakagawa H , Hosokawa M , et al. Overexpressed P‐cadherin/CDH3 promotes motility of pancreatic cancer cells by interacting with p120ctn and activating rho‐family GTPases. Cancer Res. 2005;65(8):3092‐3099. 10.1158/0008.5472.CAN-04-3646 15833838

[31] Huh YH , Oh S , Yeo YR , et al. Swiprosin‐1 stimulates cancer invasion and metastasis by increasing the Rho family of GTPase signaling. Oncotarget. 2015;6(15):13060‐13071. 10.18632/oncotarget.3637 26079945 PMC4536999

[32] Rasila T , Saavalainen O , Attalla H , et al. Astroprincin (FAM171A1, C10orf38): a regulator of human cell shape and invasive growth. Am J Pathol. 2019;189(1):177‐189. 10.1016/j.ajpath.2018.09.006 30312582

[33] Bao C , Lu Y , Chen J , et al. Exploring specific prognostic biomarkers in triple‐negative breast cancer. Cell Death Dis. 2019;10(11):807. 10.1038/s41419-019-2043-x 31649243 PMC6813359

[34] Ding X , Yang Y , Han B , et al. Transcriptomic characterization of hepatocellular carcinoma with CTNNB1 mutation. PLoS One. 2014;9(5):e95307. 10.1371/journal.pone.0095307 24798046 PMC4010419

[35] Jiang X , Zhang W , Kayed H , et al. Loss of ONECUT1 expression in human pancreatic cancer cells. Oncol Rep. 2008;19(1):157‐163. 18097590

[36] Pak E , Segal RA . Hedgehog signal transduction: key players, oncogenic drivers, and cancer therapy. Dev Cell. 2016;38(4):333‐344. 10.1016/j.devcel.2016.07.026 27554855 PMC5017307

[37] Le Borgne‐Rochet M , Angevin L , Bazellières E , et al. P‐cadherin‐induced decorin secretion is required for collagen fiber alignment and directional collective cell migration. J Cell Sci. 2019;132(21):jcs233189. 10.1242/jcs.233189 31604795

[38] Bakhoum SF , Cantley LC . The multifaceted role of chromosomal instability in cancer and its microenvironment. Cell. 2018;174(6):1347‐1360. 10.1016/j.cell.2018.08.027 30193109 PMC6136429

[39] Bakhoum SF , Ngo B , Laughney AM , et al. Chromosomal instability drives metastasis through a cytosolic DNA response. Nature. 2018;553(7689):467‐472. 10.1038/nature25432 29342134 PMC5785464

[40] Munkley J . The role of Sialyl‐Tn in cancer. Int J Mol Sci. 2016;17(3):275. 10.3390/ijms17030275 26927062 PMC4813139

[41] Julien S , Adriaenssens E , Ottenberg K , et al. ST6GalNAc I expression in MDA‐MB‐231 breast cancer cells greatly modifies their O‐glycosylation pattern and enhances their tumourigenicity. Glycobiology. 2006;16(1):54‐64. 10.1093/glycob/cwj033 16135558

[42] Ramjiawan RR , Griffioen AW , Duda DG . Anti‐angiogenesis for cancer revisited: Is there a role for combinations with immunotherapy? Angiogenesis. 2017;20(2):185‐204. 10.1007/s10456-017-9552-y 28361267 PMC5439974

[43] Ingham M , Schwartz GK . Cell‐cycle therapeutics come of age. J Clin Oncol. 2017;35(25):2949‐2959. 10.1200/JCO.2016.69.0032 28580868 PMC6075824

[44] Wardell CP , Fujita M , Yamada T , et al. Genomic characterization of biliary tract cancers identifies driver genes and predisposing mutations. J Hepatol. 2018;68(5):959‐969. 10.1016/j.jhep.2018.01.009 29360550

[45] Tiemin P , Fanzheng M , Peng X , et al. MUC13 promotes intrahepatic cholangiocarcinoma progression via EGFR/PI3K/AKT pathways. J Hepatol. 2020;72(4):761‐773. 10.1016/j.jhep.2019.11.021 31837357

[46] Urbano A , Smith J , Weeks RJ , Chatterjee A . Gene‐specific targeting of DNA methylation in the mammalian genome. Cancers (Basel). 2019;11(10):1515. 10.3390/cancers11101515 PMC6827012 31600992

[47] Chatterjee A , Stockwell PA , Rodger EJ , Parry MF , Eccles MR . scan_tcga tools for integrated epigenomic and transcriptomic analysis of tumor subgroups. Epigenomics. 2016;8(10):1315‐1330. 10.2217/epi-2016-0063 27625317

